# Graph neural networks for materials science and chemistry

**DOI:** 10.1038/s43246-022-00315-6

**Published:** 2022-11-26

**Authors:** Patrick Reiser, Marlen Neubert, André Eberhard, Luca Torresi, Chen Zhou, Chen Shao, Houssam Metni, Clint van Hoesel, Henrik Schopmans, Timo Sommer, Pascal Friederich

**Affiliations:** 1 Institute of Theoretical Informatics, Karlsruhe Institute of Technology, Am Fasanengarten 5, 76131 Karlsruhe, Germany; 2 Institute of Nanotechnology, Karlsruhe Institute of Technology, Hermann-von-Helmholtz-Platz 1, 76344 Eggenstein-Leopoldshafen, Germany; 3 ECPM, Université de Strasbourg, 25 Rue Becquerel, 67087 Strasbourg, France; 4 Department of Applied Physics, Eindhoven University of Technology, Groene Loper 19, 5612 AP Eindhoven, The Netherlands; 5 Institute for Theory of Condensed Matter, Karlsruhe Institute of Technology, Wolfgang-Gaede-Str. 1, 76131 Karlsruhe, Germany; 6 Present Address: Institute for Applied Informatics and Formal Description Systems, Karlsruhe Institute of Technology, Kaiserstr. 89, 76133 Karlsruhe, Germany; 7 Present Address: School of Chemistry, Trinity College Dublin, College Green, Dublin 2, Ireland

**Keywords:** Computational methods, Atomistic models, Method development, Computer science, Scientific data

## Abstract

Machine learning plays an increasingly important role in many areas of chemistry and materials science, being used to predict materials properties, accelerate simulations, design new structures, and predict synthesis routes of new materials. Graph neural networks (GNNs) are one of the fastest growing classes of machine learning models. They are of particular relevance for chemistry and materials science, as they directly work on a graph or structural representation of molecules and materials and therefore have full access to all relevant information required to characterize materials. In this Review, we provide an overview of the basic principles of GNNs, widely used datasets, and state-of-the-art architectures, followed by a discussion of a wide range of recent applications of GNNs in chemistry and materials science, and concluding with a road-map for the further development and application of GNNs.

## Abstract (web summary)

Graph neural networks are machine learning models that directly access the structural representation of molecules and materials. This Review discusses state-of-the-art architectures and applications of graph neural networks in materials science and chemistry, indicating a possible road-map for their further development.

## Introduction

Data science and machine learning have become an integral part of natural sciences, discussed as the fourth pillar in science, next to experiment, theory, and simulation^1^. Machine learning methods are increasingly applied in all steps of the materials development cycle, from finding initial candidate materials using property prediction^2,3^, database screening^4,5^ or even inverse materials design^6,7^, over the detailed analysis of materials in machine learning accelerated simulations^8,9^, to the prediction of synthesis conditions^10,11^ and automated experimental data analysis^12,13^ and experimental planning^14^. Machine learning models applied in chemistry and materials science cover a wide spectrum of methods, ranging from classical machine learning models such as decision tree ensembles to modern deep learning methods such as convolutional neural networks^15^ and sequence models^16^ originally developed for challenges in computer vision and natural language processing.

A recent addition to the toolbox of machine learning models for chemistry and materials science are graph neural networks (GNNs), which operate on graph-structured data and have strong ties to the field of geometric deep learning^17–19^. Aside from research on social and citation networks as well as knowledge graphs, chemistry has been one of the main drivers in the development of GNNs^20,21^. Graph neural networks can be interpreted as the generalization of convolutional neural networks to irregular-shaped graph structures. While other machine learning methods, e.g., convolutional neural networks are at the peak of publication activity, GNNs are still rising exponentially, with hundreds of papers per year since 2019. Their architecture allows them to directly work on natural input representations of molecules and materials, which are chemical graphs of atoms and bonds, or even 3D structures or point clouds of atoms. Therefore, GNNs have access to a complete representation of materials on the atomic level^22^, with a lot of flexibility to incorporate physical laws^23^, as well as phenomena on larger scales, such as doping and disorder. Using that information, GNNs can learn internal materials representations that are useful and informative for specific tasks such as the prediction of given materials’ properties. Therefore, GNNs can complement or even replace hand-crafted feature representations which were and are widely used in the context of natural sciences in general. A similar trend toward representation learning methods has also been observed in other application areas during the last years, where end-to-end trainable models show a systematic advantage over traditional feature-based methods^24^. However, despite promising recent developments toward higher sample efficiency^25,26^, this often comes at the cost of higher data requirements^27^, potentially limiting the applicability of existing GNNs to applications where large amounts of data are available. Overall, GNNs outperformed conventional machine learning models in predicting molecular properties throughout the last years^22,28,29^. While GNNs are not as widely applied (yet) in materials science as they are in chemistry, there are advantages and the potential to outperform other machine learning methods and thus boost virtual materials design and materials science in general, which will be discussed in this article.

In section ‘Graph neural networks in materials science and chemistry’, we will introduce the general formalism of GNNs and discuss the way they transform the atomic structure of materials and molecules and use it to predict materials’ properties. We will present and compare state-of-the-art architectures and benchmark datasets, as well as summarize initial efforts toward inverse materials design based on GNNs. The section ‘Applications’ covers a wide range of current application areas but also open challenges for GNNs in chemistry and materials science. In section ‘Outlook’ we conclude with a perspective on necessary and expected future developments and so far unused potential of GNNs in materials science.

## Graph neural networks in materials science and chemistry

### Basic principles

In the most general sense, graphs are used to describe abstract structures consisting of entities or objects represented as vertices (or nodes) and their connections, called edges. Formally, a graph is a tuple *G* = (*V*, *E*) of a set of vertices *v* ∈ *V* and a set of edges *e*_*v*,*w*_ = (*v*, *w*) ∈ *E*, which defines the connection between vertices. Potential tasks that can be solved using graph neural networks (GNNs) include classification or regression of graph properties on graph level (molecular property prediction), node level (classification of members, i.e., nodes, of a social graph), or edge level (prediction of relations, i.e., edges, between objects in a scene graph). In materials science and chemistry, most tasks involve graph-level predictions, which will be the focus of this paper.

The concept of graphs is used in mathematical chemistry to represent the structure of compounds. The molecular structure is represented by an undirected graph, where nodes correspond to atoms and edges correspond to chemical bonds. In fact, chemical graphs were first considered as early as in 1874^30^ and their idea traces back further^31^, which may place them even before the advent of the term graph in modern graph theory^32^. The description of molecules as graphs can also be transferred to solid-state materials, even though bonds might not be uniquely defined in crystals, and the exact three-dimensional arrangement of atoms plays a more decisive role.

Since their proposal^17–19^, GNNs have become a popular machine learning method for processing irregularly shaped data encoded as graphs. They can be seen as an alternative to approaches, where predefined feature representations of molecules or materials are used as input to conventional machine learning models such as densely connected neural networks, random forest models, or Gaussian process regression models. In the case of GNNs, the full molecular structure or even geometry is used as input and the GNN itself learns informative molecular representations to predict given target properties. Due to their popularity and wide applicability, a large number of different GNN architectures have been proposed^18,20,21,33–35^. While the exact architecture type can notably differ, ranging from the initially proposed recursive GNNs^18^ to spectral neural filters^35,36^ and finally to spatial or convolutional GNNs^34^, most GNNs designed for chemistry and materials science can be summarized under the framework of Message Passing Graph Neural Networks (MPNN) as suggested by Gilmer et al.^21^. In this section, we give an overview of ideas of the message passing framework and discuss how learned graph- or node-level embeddings can be used for materials property prediction.

**Fig. 1:**
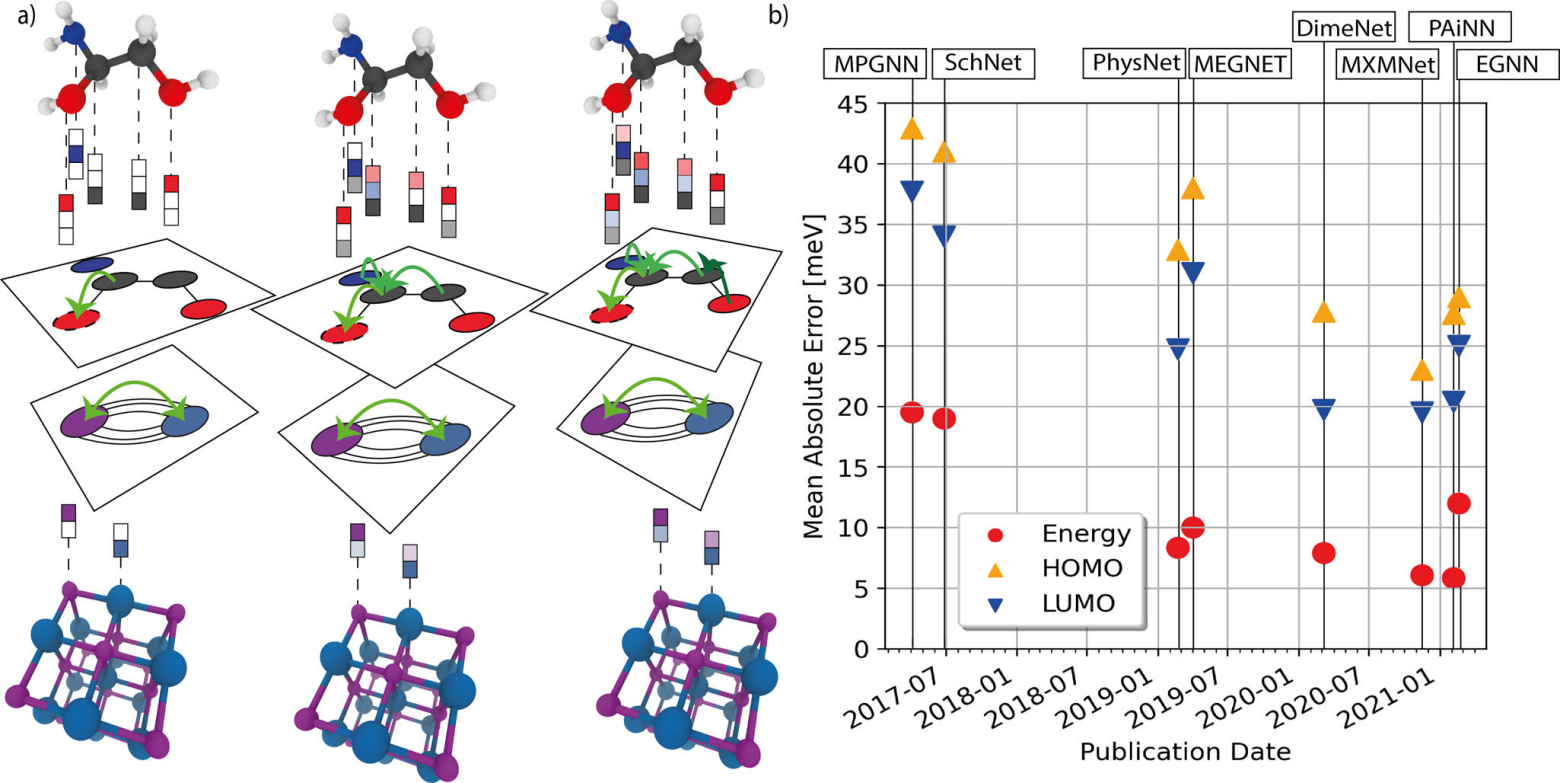
Overview of the message passing principle and performances of GNNS. **a** Schematic depiction of the message passing operation for molecules and crystalline materials. **b** QM9 benchmark. Mean absolute error of the prediction of internal (red circles), highest occupied molecular orbital (HOMO, orange triangles), and lowest unoccupied molecular orbital (LUMO, inverted blue triangles) energies for different GNN models since 2017.

For MPNNs, associated node or edge information (e.g., atom and bond types) is commonly provided by node attributes ${h}_{v}^{0}\in {{\mathbb{R}}}^{d}$ and edge attributes ${h}_{e}^{0}\in {{\mathbb{R}}}^{c}$. Details about feature and structure representations are discussed in Section ‘Structure representation’. Using node and edge features in combination with the graph’s structure, GNNs are capable of deriving a node-level embedding of the graph, i.e., learned vectors representing each atom including its individual chemical environment. This is done in the so-called message passing phase, in which node information is propagated in form of messages *m*_*v*_ through edges to neighboring nodes. The embedding of each node is then updated based on all incoming messages. The locality of the message passing is sought to be alleviated by repeating the message passing phase *t* = 1…*K* times, in principle allowing information to travel longer distances, i.e., within the K-hop neighborhood. In practice, however, information from long-range dependencies can be distorted in node bottlenecks, referred to as over-squashing^37^, or be washed out, leaving indistinguishable representations of neighboring nodes, known as over-smoothing^38^. Note that for typical (not fully linear) molecules and crystal unit cells with *n* atoms, only approximately $\log n$ message passing steps are required to pass information to all other atoms. The information processing is facilitated by the learnable functions *U*_*t*_(⋅) for node update and *M*_*t*_(⋅) for the message generation. Finally, in the readout phase, a graph-level embedding *y* is obtained by pooling node embeddings of the entire graph via a parametric readout function *R*(⋅). The final representation of the graph is used for training both regression and classification tasks. In summary, the MPNN scheme reads^21^:

1 $${m}_{v}^{t+1}=\mathop{\sum}\limits_{w\in N(v)}{M}_{t}({h}_{v}^{t},{h}_{w}^{t},{e}_{vw})$$

2 $${h}_{v}^{t+1}={U}_{t}({h}_{v}^{t},{m}_{v}^{t+1})$$

3 $$y=R(\{{h}_{v}^{K}| v\in G\}),$$

where *N*(*v*) = {*u* ∈ *V*∣(*v*, *u*) ∈ *E*} denotes the set of neighbors of node *v*. Note that readout and aggregation can be in principle any mathematical operation that is permutation invariant, e.g., a sum, mean or maximum operation similar to Eq. (1) or learnable such as the Set2Set encoder proposed by Vinyals et al.^39^, which was originally used for the readout *R*(⋅). The learnable functions are mostly neural networks and eventually determine the performance characteristics of the GNN, both in prediction accuracy and computational cost. Figure 1a shows a schematic of the message passing scheme for the example of a molecular graph. Message passing can also be understood as a convolution operation running over each node in a graph. Different extensions and modifications of the message passing schemes are discussed in Section ‘State-of-the-art architectures and benchmarks’ and include edge updates^40^, skip connections^41^, and geometric information^26,42,43^.

A main open research question of GNNs revolves around their limited expressive performance for specific tasks^44^ and how GNNs compare with Weisfeiler–Lehman hierarchy for graph isomorphism testing^45,46^. With regard to this topic, there are many promising extensions to GNNs proposed in literature, such as hypergraph representations^47,48^, universal equivariant models^49^ or higher-order graph networks^50^. Furthermore, the challenge of over-smoothing due to commonly used aggregation functions^38^, transfer and multitask learning^51^, as well as training (in)stability^52^ are subject of current research.

### Structure representation

Many graph networks directly use the chemical graph as input, representing both molecules^21^ and inorganic compounds^24,53^, and offering advantages over compositional or fixed-sized vector representations in terms of flexibility and scalability. Consequently, GNNs can be applied for tasks such as drug design or material screening^54^, which require knowledge about functional groups, scaffolds^55^ or the full chemical structure and its topology. In molecular applications, the chemical graph is often extracted from SMILES codes and augmented with features that can be readily obtained from cheminformatics software such as RDKit^56^ or OpenBabel^57^. Common features for atoms, bonds, and the molecule itself are listed in Table 1. Besides hand-crafted input features, learned embeddings of molecules and materials motivated by word embedding techniques in natural language processing have been explored which can be used for downstream tasks^48,58–60^. For specific tasks in chemistry, the connectivity of atoms in molecules (i.e., the molecular graph) contains sufficient and complete information to predict given molecular properties which do not depend on the exact geometry. Geometry or stereochemical information can be taken into account e.g., in form of additional edge features representing the distance between atoms^61^. In contrast to that, in materials applications, atom connectivity is not well defined in most cases (apart from e.g., covalently linked frameworks) and graphs have to be extracted from crystal structures based on distance heuristics.

**Table 1:** Table of typical (molecular) graph features used in literature^54,269^.

Graph-level	Attributes	Description
nodes	atom-type	type of atoms (one-hot)
	chirality	R or S (one-hot or null)
	degree	number of covalent bonds (one-hot)
	radical	number of radical electrons (integer)
	hybridization	sp, sp^2^, sp^3^… (one-hot)
	aromaticity	part of an aromatic system (binary)
	charge	formal charge (integer)
edges	bond-type	single, double, … (one-hot)
	conjugation	is conjugated (binary)
	ring	bond is part of a ring (binary)
	stereo	None, Any, Z, E (one-hot)
graph	weight	average atomic weight (float)
	bonds	average bonds per atom (float)

They can be further combined with geometric features^61^.

The sole chemical graph and its connectivity are often not sufficient to accurately predict quantum-mechanical or electronic-structure properties^62^ that strongly depend on the exact molecular geometry, even though ground-state or equilibrium geometries can in principle be inferred from the molecular graph alone. In tasks that intrinsically involve geometric dependencies, e.g., predicting the potential energy surface of molecules and materials, it becomes obvious that geometric information is required. The representation of positional and geometric information to learn quantum properties has been explored among others in the work of Lilienfeld et al.^63^ and Behler et al.^64^ and lead to a large variety of descriptors. Some examples of descriptors are atomic centered symmetry functions (ACSF)^65,66^, angular Fourier series (AFS)^67^, the smooth overlap of atomic orbitals (SOAP)^67^, partial radial distribution function (PRDF)^68^, many-body tensor (MBTR)^69^, Spectral London Axilrod-Teller-Muto (SLATM)^70^ and the Faber-Christensen-Huang-Lilienfeld (FCHL)^71^ representation. Many of those descriptors expand geometric information into symmetry or basis functions. The resulting vector representation is typically used as input for conventional machine learning models such as neural networks or Gaussian Processes. Geometric information can also be used for node or edge representations in graph neural networks. Graph networks have been adopting distances^61^, bond^72^ and even dihedral angles^26,73^, motivated by the comparison to force fields^74^. Angles or distances are similarly expanded into Gaussian-like^22^, radial^23^ and spherical Fourier-Bessel functions^28^. Although architectures such as the Behler-Parinello (BP) neural network potentials^8^ or SchNet^22^ are not strictly graph networks in terms of the chemical graph, and often do not refer to themselves as such, they can be summarized within the term geometric deep learning^75,76^.

Under the term geometric deep learning, architectures and descriptors are summarized that focus on manifolds, geometric data, or structured data^77,78^. This includes the work on 3D point clouds^79^, which aims at learning segmentation and object detection of a large number of 3D points. In the case of PointNet++^80^ a graph is constructed which reduces the point set from learned descriptors using the points’ features. Commonly, adjacency matrices are defined by using distance cutoffs between points in 3D clouds, while edges carry explicit information about distances between nodes, i.e., points. Graph pooling or coarsening algorithms^81,82^ that reduce the input representation and condense structure information are also promising for GNNs to tackle larger molecules such as proteins or polymers.

Eventually, the representation of materials for graph networks can be structural or geometric but must follow certain symmetry considerations^83,84^. For example molecules without external fields have rotational and translation symmetries. If they are incorporated into the model and its representation, less data are required and overall performance can be improved. This concept can be extended to equivariant representations^85,86^, which in combination with equivariant filters, enable equivariant graph neural networks^29,87^. Scalar features are extended to (directional) features, like vectors and higher-order tensors (in the geometric context of the term tensor), since, embedded in 3D Euclidean space, they can transform predictably under geometric rotation, reflection, and translation^25,87^.

For solid crystals and periodic structures, the periodicity and space group symmetries are additional symmetries to be added to the representation for GNNs. Periodic extensions of the crystal graph^2,53^ of the unit cell have been introduced^61^ and their representation builds on the idea of a k-point mesh of Monkhorst-Pack grid points to sample the Brillouin zone^88^.

### State-of-the-art architectures and benchmarks

Different architectures have been proposed in the literature to improve the performance of GNNs on typical tasks arising in chemistry and materials sciences. Table 2 shows a list of popular benchmark datasets for different materials classes, i.e., molecules^62,89–99^ or crystals^100–102^, and respective supervised tasks, i.e., regression or classification. While some datasets contain experimental data, the largest datasets typically use computational methods to generate labels. Most datasets in this table can be downloaded from data collections such as TUDatasets^103^ and MoleculeNet^95^. While a large variety of datasets and tasks exist for chemistry, there are only a few large datasets for materials, limited to crystalline structures. Recent datasets were constructed by filtering the Materials Project (MP)^100^ and Open Quantum Materials Database (OQMD)^101^ for specific targets such as electronic band-gap or formation energy while removing incomplete data^88^.

**Table 2:** Table of common benchmark datasets for graph learning tasks.

Molecules	Size	Tasks	Type	Description
QM7^89^	7165	1	R	DFT quantum calculations
QM7b^90^	7211	13	R	DFT quantum calculations
QM9^62^	133,885	12	R	DFT quantum calculations
PDBBind^91^	23,496	1	R	protein binding affinity
MD17^92,93^	>100,000	≥1	R	molecular dynamics trajectories
FreeSolv^94^	643	1	R	solvation free energy
Lipop^95^	4200	1	R	lipophilicity
Tox21^95^	8014	12	C	qualitative toxicity measurement
ToxCast^96^	8615	617	C	qualitative toxicity measurement
BBBP^97^	2053	1	C	blood–brain barrier penetration
HIV^95^	41,913	1	C	inhibition to virus HIV
SIDER^98,99^	1427	27	C	adverse drug reaction

Crystals	Size	Tasks	Type	Description
MP^100^	~144,595	≥1	R, C	Materials Project (MP)
OQMD^101^	~1,022,603	≥1	R, C	Open Quantum Materials Database
OC20^102^	~133,934,018	≥1	R	Open Catalyst Project

Note that this list is not complete and merely serves as an overview of different sizes and supervised learning tasks, which is either regression (R) or classification (C).

In Section ‘Basic principles’, the message passing framework for GNNs has been illustrated. Here, we will discuss modified and extended GNN models, which are relevant for materials science and chemistry. However, listing all graph network architectures would be beyond the scope of this review.

Some of the earliest work on neural networks for molecular graphs dates back to the 90s and 2000s, without explicitly referring to the term graph neural network^8,33^. In 2017, a graph convolutional network was proposed by Kipf et al.^34^ for semi-supervised learning, which can be interpreted as a first-order approximation of spectral graph convolutions^35,36^. In Table 3, we distinguish GNN architectures that operate on the graph’s spectrum in category spectral convolution and GCN models that process the structure of the graph in spatial convolution, which in principle includes message passing schemes that pass information along edges in the graph. We added a soft distinction in this category to separate models that follow the convolution naming and models that explicitly refer to the term message passing and its extension.

**Table 3:** Table of GNN models are sorted by categories.

Categories	GNN architectures
Spectral convolution	LanczosNet^270^, SpecConv^271,272^, CayleyNet^273^, ChebNet^35^
Spatial convolution	GCN^34^, 123-GNN or k-GNN^50^, R-GCN^274^, GIN^46^
	PatchySan^275^, C-SGEL^276^, GraphSAGE^106^, OGCNN^2^
	CGCNN and iCGCNN^225,233^
Message passing	MPNN^21^, D-MPNN^40^, MPSN^44^, MGN^33^
	G-MPNN and MPNN-R^277^, PMP^278^
3D geometric message passing	MEGNET^61^, DimeNet^28,72^, PhysNet^23^, MolNet^83,279^
	PointNet++^80^, MXMNet^111^, SchNet^22,109^, ForceNet^191^,
	GemNet^26^, Geomol^113^, ALIGNN^110^ and ALIGNN-d^112^,
	GNNFF^190^, GeoCGNN^88^, SphereNet^280^, HGCN^119^
Attention and graph transformer	GAT^108^, GATv2^281^, MAT^51^, AGNN^282^, AMPNN^283^
	CapsGNN^284^, RGAT^285^, AttentiveFP^54^, AGN^52^
	GACNN^184^, MEGAN^130^, SAMPN^286^, HamNet^287^
Equivariant models	PaiNN^29^, NequIP^25^, TFN^87^, CGNet^288^,
	Cormorant^114^, LieConv^43^, EGNN^85^, UNiTE^115^
	SEGNN^289^, SE(3)T^290^, CNN-G^291–293^
Graph pooling	DiffPool^294^, EdgePool^295^, gPool^296^
	HGP-SL^82^, SAGPool^81^, iPool^297^, EigenPool^298^
Generative graph models	CGVAE^146^, JT-VAE^147^, GCPN^154^, GeoMol^113^
	GraphGAN^151^, DCGAN^150^

It is to note, that some models can also fall into more than one category and that this table can not list all relevant models but only give a grouping of a few popular models mentioned in the text. There is no strict distinction between categories spatial convolution, message passing, and 3D geometric message passing. Generative models are discussed in Section ‘GNNs in generative models’.

The addition of more complicated node aggregation functions such as gated recurrent units^104^ or long short-term memories^105^ has been employed by GraphSAGE for inductive learning^106^. For graph embedding tasks, a state- or super-node^21^, which is connected to all nodes, extends the message passing framework to help extract global graph information, in addition to the final node aggregation step. A message passing neural network (MPNN) with edge features capturing bond information was applied to molecular graphs^21^ and crystal graphs^53^. A variant of the original MPNN involves directed edge embeddings and message passing between edges in D-MPNN^40^. Known from models in natural language processing^107^, masked self-attention layers which attend over the node’s neighborhood have been suggested for graph attention networks^108^ and used explicitly for molecules in Attentive Fingerprint models^54^. More attention-based architectures are listed in Table 3.

Besides graph models which focus on the chemical graph, there is a large group of models explicitly designed for learning quantum properties. They commonly take atomic numbers and positions as input and train on data derived from (approximate) solutions of the steady-state Schrödinger equation. A popular benchmark dataset is the QM9 dataset^62^ with 13 quantum properties of small molecules with up to nine atoms apart from hydrogen. The improvement of graph networks on QM9 property prediction over the past few years is highlighted in Fig. 1b. Among the first graph networks that reached chemical accuracy on QM9 is SchNet^22^, which makes use of convolutional filters for inter-atomic distances and applies skip connections between node updates. One improvement to SchNet was to update the positional features along the graph edges as seen in Jørgensen et al.^109^. The application of GNNs to crystals using geometric information has been explored by MEGNET^61^, which further leverages global properties such as temperature, which is of importance for solid-state crystalline systems. The potential energy of molecules depends on bond angles and therefore, in DimeNet^28,72^, edge embedding uses messages passing steps from atomic triplets and bond pairs in order to incorporate angular features. This formalism has been adopted in other recent GNNs^110,111^ and can be further extended to include dihedral (or torsion) angles^26,112,113^.

For explicit angle plus node information in directed edge updates as in DimeNet^28,72^, the message passing essentially operates on higher order paths^73^ or *k*-pairs of atoms^50^. This is unfeasible for fully connected larger graphs because the number of multi-node interactions that need to be computed is dramatically increasing. To reduce the computational costs, models like MXMNet^111^ make use of multiplex graphs, which selectively consider only specific edges when going to higher order pathways for calculating bond angles^110^.

Note that the GNNs mentioned previously are invariant to the translation and rotation of the molecules throughout space. Recently, equivariant GNNs have been proposed^29,43,85,114^, which transform equivariantly under symmetry operations of its (positional) input, meaning the GNN’s features or its output undergoes the same operations as well. The type of symmetry operations is commonly specified in 3D-space by the corresponding Euclidean group E(3). The special (isometric) subgroup of E(3), which preserves handedness in transformation, is denoted by SE(3). Symmetry operations preserving distance and a fixed point form the orthogonal euclidean group. Analogously, the orthogonal group is denoted by O(3) and its composable special group by SO(3). For example, SO(3) contains rotations, O(3) includes rotations plus reflections, SE(3) allows rotations plus translations, and E(3) encompasses both rotations, translations and reflections. For GNNs also the permutation equivariance with respect to the input set of nodes can be considered and is characterized the by symmetric group S_*n*_. In TFN^87^ equivariant filters are constructed based on learnable radial functions and spherical harmonics for (geometric) feature vectors of different order, namely scalars (type-0), vectors (type-1) and higher-order tensors, which comprise a direct sum of irreducible representations of the O(3) symmetry group. Equivariant convolution layers can be obtained by tensor-products thereof using Clebsch-Gordan coefficients^87^. Simplifying to type-1 representations, EGNN^85^ uses the relative squared distance and updates the position of each particle as a vector field in a radial direction to preserve E(3) equivariance. Equivariant graph models convey directional information between atoms^29^ without higher-order pathways and enable the prediction of tensorial properties^114,115^. We have listed popular equivariant GNNs is a separate category in Table 3.

Further adapted message passing steps allow for the determination of the molecular orbitals^2,116–118^. Molecular orbital interactions can in turn be used for improving the prediction performance^2^. Lastly, the mapping of atoms to non-Euclidean space such as in the proposed hyperbolic GNNs^119^ can lead to gains in representational efficiency. For a more in-depth discussion of graph variants^120^ and graph taxonomy^121^ that goes beyond Table 3, we refer to more general articles about GNNs^122,123^, e.g., Zhou et al.^121^ and Wu et al.^124^.

With regard to the QM9 benchmark in Fig. 1b some models have slightly lower performance for the total energy but can be superior in other QM9 properties or achieve similar results with much less computational effort. Some other factors that complicate a stringent comparison are differences in train-test splits, cleaning steps, e.g., of ill-converged molecules in QM9, multi-task vs. single task settings, where a separate model for each QM9 target is usually trained^61^, and differences in used loss metrics (a mean absolute error loss was found to yield lower overall test errors^23^ than the mean squared error loss used in previous models^22^, although the mean absolute error is typically given as a benchmark reference). It has to be noted that hyperparameters are generally very important and are often not exhaustively optimized for GNNs which can cause differences in performance apart from the model architecture^125–127^.

### GNNs in generative models and reinforcement learning

An important challenge in materials science is inverse materials design, aiming to generate new materials or molecules that possess required properties and fulfill specific criteria^128^. GNN-based generative methods have been suggested to deal with this challenge in the context of chemistry, e.g., for drug discovery^129^ and retrosynthesis^130^. In most cases, only the chemical structure of molecules is generated, i.e., the connectivity of the molecular graph, without additional information on specific 3D geometry.

Initial graph generative models were designed to generate graphs based on simplified theoretical assumptions, such as the random Erdös-Renyi (ER) model^131^, and improvements thereof using small-world approaches^132^ or the Kronecker graph model^133^. While these traditional approaches attempt to model real-world graphs, they are based on several assumptions about the nature of graphs generated and are thus inflexible for many data-related applications. Machine learning approaches for graph generation are promising because they can directly learn to generate realistic graphs from the distribution of observed data while accommodating goal-directed tasks such as property optimization. Examples include variational autoencoders (VAEs)^134^, generative adversarial networks (GANs)^135^, reinforcement learning^136^, recurrent neural networks (RNNs)^137^, and flow-based generative models^138–140^.

Several architectures of VAEs have been developed to work with different types of input data, such as images^141^, text-based data^142,143^, or graphs^144,145^. Kipf et al. introduced a variational graph auto-encoder (VGAE) to learn latent representations of undirected graphs and applied it to a link prediction task in citation networks^144^. Liu et al. introduced a Constrained Graph Variational Autoencoder (CGVAE), in which node type information is learned from and added to the latent vectors^146^. Starting from a set of these latent node representations, CGVAE iteratively forms valid molecules following hard valency constraints derived from the explicit node types. Jin et al. introduced Junction Tree Variational Autoencoder (JT-VAE) to work directly on molecular graphs and achieved an improvement over baseline methods in molecular design tasks^147^. The JT-VAE approach encodes and decodes molecules in two steps: First, tree-structured objects called junction trees are generated which represent trees of molecular subgraphs and their arrangements. GNN-based encoders and decoders are used to generate latent embeddings of the junction trees. In parallel, molecular graph embeddings are generated using GNNs, and junction trees are decoded into molecular representations^21^. These are then encoded to a latent vector and decoded back to their original representations using graph and tree-based encoders and decoders. While this scheme works well for molecules, it is hard to adapt for crystalline materials, where the graphs are less tree-like and the definition of scaffolds is not as straightforward.

GANs have shown promising results in a number of fields, such as image^148^ or sequence^149^ generation, and have also been applied to 3D grid representations of materials^150^ and graphs^151^. De Cao et al. introduced MolGAN^152^ as a framework for generating molecular graphs using GNNs. The generator learns to directly output the graph’s representation. While the standard GAN loss forces the generator to generate molecules following a particular prior distribution, the authors add a reinforcement learning (RL) objective to generate molecules with optimized properties. The generation of invalid molecules is avoided by the assignment of zero reward to them. While direct prediction of outputs is appealing in methods using VAEs or GANs, they usually predict outputs of small, fixed sizes. Therefore, another branch of deep graph generative models employs sequential decision-making procedures to overcome these limitations.

You et al. identify three challenging aspects when graphs are generated directly^137^. First, as these methods need to model the adjacency matrix in some form, the output size grows as ${{{{{{{\mathcal{O}}}}}}}}({n}^{2})$ for graphs with a maximum number of *n* nodes. This is especially undesirable for large and sparse graphs as the model dedicates much of its capacity to learn which nodes are not connected. Second, graph isomorphism complicates the calculation of reconstruction losses for VAEs and usually involves expensive graph matching procedures. Third, VAEs assume the outputs, e.g., the entries of an adjacency matrix, to be i.i.d., which is not true in practice. As a solution, the authors propose GraphRNN, a framework in which graphs are represented as sequences of node and edge additions. By using recurrent networks, graph constituents are generated conditioned on previous additions, thus taking into account the history of modifications. Another sequential graph generation scheme was proposed by Li et al.^153^. In this framework, the generation process is decomposed into modular steps, e.g., whether to add a node or which nodes to connect. Each module is a fully-connected neural network modeling probabilities of executing particular types of modifications.

**Fig. 2:**
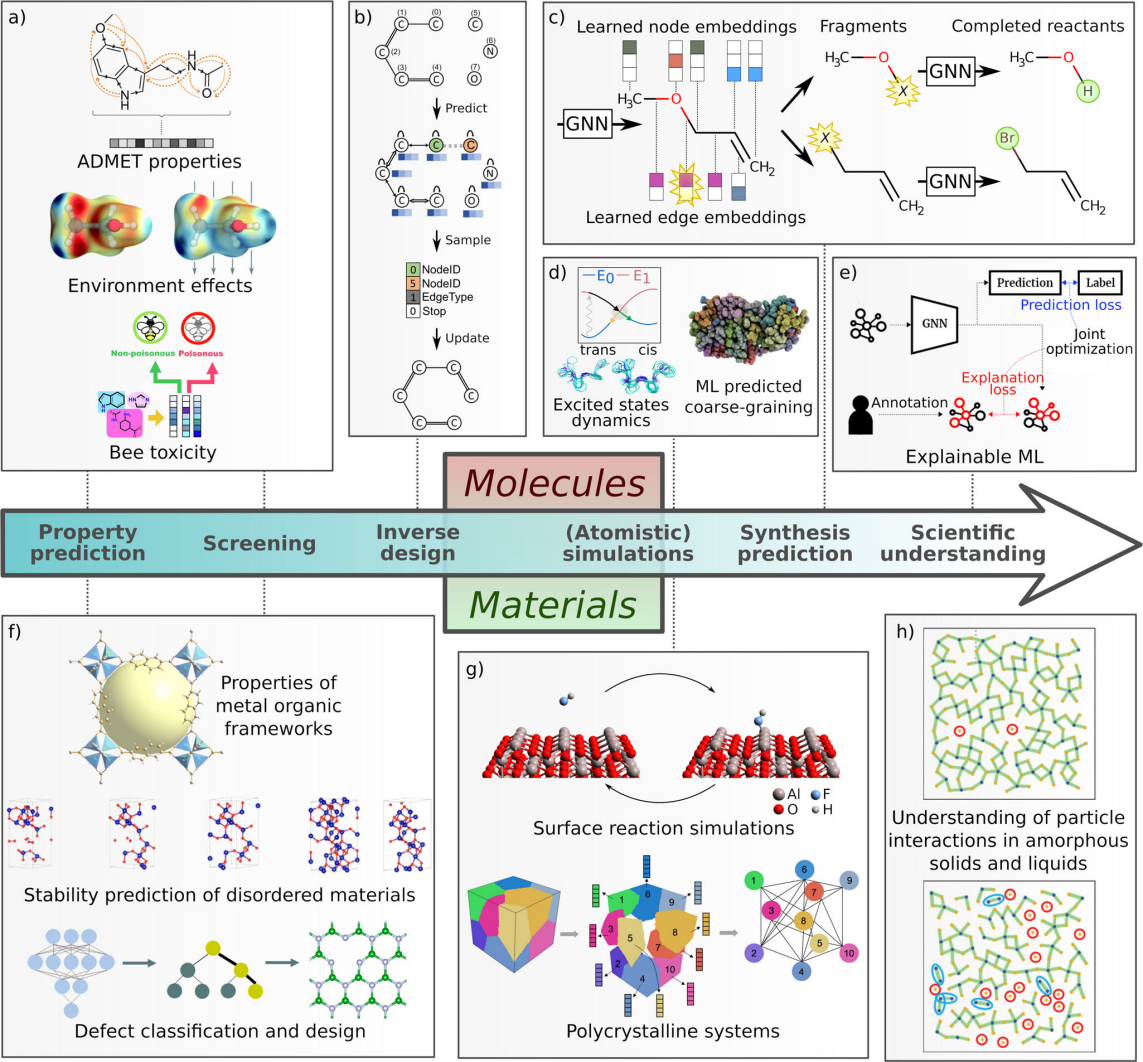
Overview of GNN applications for molecules and materials. **a** prediction of ADMET properties (adapted with permission from Feiberg et al.^165^, Copyright 2020 American Chemical Society), GNNs accounting for environment effects of molecules (reproduced from ref. ^179^ with permission from the Royal Society of Chemistry.), GNNs to predict the toxicity of molecules for bees (this illustration was published in ref. ^184^, Copyright Elsevier), **b** RL-based approach for inverse molecular design based on Graph Convolutional Policy Networks (GCPN) (adapted from ref. ^154^), **c** template-free retrosynthesis (adapted from ref. ^221^), **d** transferable excited states dynamics (reproduced from ref. ^199^ with permission from Springer Nature), coarse graining (reproduced from ref. ^194^ with permission from the Royal Society of Chemistry), **e** explainable GNNs (adapted from ref. ^311^), **f** Crystal GNN to predict methane adsorption volumes in metal organic frameworks (MOFs) (this illustration was published in ref. ^234^, Copyright Elsevier), doped structures (this illustration was published in ref. ^244^, Copyright Elsevier), point defects (adapted with permission from Frey et al.^245^, Copyright 2020 American Chemical Society) **g** reactions of Al_2_O_3_ surface in contact with HF gas (reproduced from ref. ^190^ with permission from Springer Nature), GNNs to predict magnetostriction of polycrystalline systems (reproduced from ref. ^240^ with permission from Springer Nature), **h** a GNN classifier to predict if a system is in a liquid or a glassy phase only by the positions of the atoms (reproduced from ref. ^249^ with permission from the Royal Society of Chemistry).

You et al. suggested a purely RL-based approach based on Graph Convolutional Policy Networks (GCPN)^154^ (see Fig. 2b). In this setting, the agent explores the chemical space and generates new molecular graphs by modifying the starting molecules according to the reward function, representing the molecular property to optimize. Atance et al. recently introduced a similar RL approach based on Gated GNNs^155^, outperforming other GNN-based approaches in molecular graph generation tasks^156^. Another sequential approach based on conditional graph generative models has been used by Li et al. on drug design tasks^157^. The previous two works inspired recent molecular graph generation frameworks such as GraphINVENT^156^.

More recently, attention has turned to generative modeling based on normalizing flows (NFs)^158^, capable of modeling complex target distributions by directly mapping them to simpler ones using revertible bijective transformations. NFs potentially offer significant improvements in the field of graph generation because they allow exact evaluation and inference of the probability distribution. This approach has been applied in a number of molecular tasks^129,159–161^.

Overall, graph generative models have been extensively applied for molecular materials and stayed up to date with recent developments in the field of graph generation. However, these remain under-explored for crystalline materials, mainly due to graph representation challenges^162^. While finding such a reliable graph representation is still an open question^128^ and will likely remain case-specific, we believe that using generative models based on GNNs is a promising research direction in inverse design, especially given current breakthroughs such as normalizing flows.

## Applications

After introducing the basic principles of GNNs as well as selected GNN architectures and benchmark datasets, we will provide a structured overview of GNN applications in chemistry and materials science. GNNs were successfully applied to a rich variety of different challenges, ranging from property prediction of molecules and materials over accelerated atomistic simulations to predicting reactivity and synthesis routes of molecules and materials. While other machine learning models such as densely connected neural networks were successfully applied to these tasks as well, state-of-the-art GNNs in many cases currently outperform other models. However, there exists a range of open challenges, including data requirements and data efficiency, as well as a lack of fully GNN-based generative models for molecular and materials design.

### Molecular systems

Some of the first applications of GNNs and probably also one of the main driving forces for the ongoing development of GNN models are challenges in the area of molecular chemistry. Most prevalent is the task of predicting molecular properties, i.e., a regression or classification task which is challenging to solve with conventional machine learning models, as they typically require predefined molecular representations (e.g., molecular feature vectors or molecular fingerprints) which are informative for the label to predict. GNNs have access to the full chemical graph or even molecular geometry and learn to extract feature representations, which yields an advantage over other machine learning models. Compared to domain knowledge-informed feature representations combined with conventional machine learning models, e.g., Gaussian process regression, GNNs often have comparably high data requirements but outperform conventional models when enough data is available. Once trained, accurate machine learning models can then be used to accelerate the high-throughput virtual screening of molecules^163,164^ to find promising candidate molecules for many different applications. However, property prediction is not the only application of GNNs. They were also successfully applied to provide trainable interatomic potentials to accelerate costly ab initio molecular dynamics simulations, as well as to predict the outcome of chemical reactions and synthetic routes.

#### Molecular property prediction

Among the most relevant molecular properties in the area of drug discovery are the ADMET (absorption, distribution, metabolism, exclusion, and toxicity) properties of potential drug-like molecules (see Fig. 2a)^165–168^. A review on GNNs for drug design can be found in Xiong et al.^169^. In recent years, one application focus were Covid 19 related challenges, where GNNs were used for e.g., finding new drug candidates^170^ or detecting infections in medical images^171,172^. Similar methods are also applicable to other challenges in drug design and medicine.

Furthermore, GNNs were applied to predict electronic and optical properties of molecules. For many applications such as organic electronics, organic photovoltaics, and organic light-emitting diodes, the energy of the highest occupied molecular orbital (HOMO), the lowest unoccupied MO (LUMO), and the optical gap are of high importance for the device efficiency. These properties can therefore be found in numerous databases^62,90,173–177^. Related properties include (transition) dipole moment^62,178^, ionization potential, and electron affinity^90^. In devices, these properties often depend on the molecular environment, which can be modeled and accounted for with GNNs (see Fig. 2a)^179^. For certain applications such as opto-electronic materials^174,175^, GNNs have been proposed to complement scalar molecular properties with spectroscopic properties^112^.

The aforementioned properties are often determined using computationally expensive simulation methods. Cheaper, mostly semi-empirical methods can provide fast but potentially less accurate estimates. GNNs have been used to represent Kohn-Sham wavefunctions and energy levels in a minimal basis representation^180^, as well as for delta-learning from semi-empirical methods to DFT computed molecular properties^177^. For applications where not enough data is available to train GNNs, the representations learned by GNNs on large generic datasets can also be transferred to supervised tasks with little data^20,181,182^, where they are used as input for other machine learning models such as gradient-boosted models^183^.

Further application areas of GNNs spread across all application domains of molecular materials, including the prediction of toxicity of molecules to bees (see Fig. 2a),^184^ determining the quality of a material for fuel ignition^185^ and the classification of different phases of materials, in particular water^186^. It should be noted that another review paper on molecular property prediction utilizing GNNs exists by Wieder et al.^120^. In many application areas, tools such as GNNExplainer^187^ are used to validate and analyze GNN predictions, e.g., in the prediction of scents^188^ and for porous organic cages^189^.

#### Dynamics simulations

Molecular dynamics simulations are an important tool for understanding dynamic processes and mechanisms on a microscopic level in various areas of chemistry, biology, and materials science. Besides the prediction of equilibrium properties of molecules and materials, they also offer the possibility to simulate excited states and non-equilibrium dynamics, as well as slow processes and rare events.

In molecular dynamics simulations, total energy and forces are needed in every time step to propagate the system. Computationally demanding ab initio methods that calculate the energy and forces of a particular atomic configuration of a system at every time step are therefore often too costly. Machine learning methods can replace ab initio calculations to speed up simulations while ideally retaining their accuracy^9^. Therefore, long and highly accurate MD simulations can be performed based on machine-learned potentials, which have not been possible using classical force fields nor ab initio methods. GNNs are perfectly suited for this task, as atomic forces depend on the (local) atomic environment and global aggregation is not needed.

The concept of integrating machine learning models in atomistic simulations was demonstrated multiple times using for example SchNet^22^, PhysNet^23^, DimeNet^28^, or DimeNet++^72^. However, there are several open challenges that need to be overcome in order to move to larger systems, longer time scales, higher data efficiency, better generalization and transferability, and eventually more accurate and realistic applications. Usually, machine learning models learn the potential energy surface and calculate forces using derivatives of the energy predictions. This ensures that energy predictions and forces are consistent. Since only forces are required in MD simulations, architectures are being developed in which these forces are predicted directly—so that the costly derivative calculations are omitted. In the GNN framework (GNNFF^190^), a message passing step builds upon an embedding step in which node and edge features include atom type and interatomic distances respectively. Force magnitudes per atom are then calculated from the sum of the forces of the neighboring atoms. Evaluation on the ISO17 database reveals higher force accuracies compared to SchNet while being 1.6 × faster. The approach is also shown to be scalable to larger systems. Due to the direct prediction of the forces, the energy of the system is however not necessarily preserved, making the model not suitable to predict energy-related properties.

ForceNet^191^ is based on an encoder-decoder architecture and tries to completely capture 3D geometry through a specific design of the message passing structure. In contrast to models such as SchNet and DimeNet, ForceNet encodes physical information without constraining the model architecture to enforce physical invariances. Instead, physics-based data augmentation is performed on the data level to achieve rotational invariance. The evaluation was performed on the OC20 dataset which contains DFT calculated energies and per-atom forces of more than 200 million large atomic structures (20–200 atoms) including non-equilibrium structures from optimization trajectories. The resulting mean absolute force errors are comparable to DimeNet++, while being faster in training and prediction.

A promising approach to encoding more physical information about a system is the design of equivariant models. Models that are based on equivariant message passing, e.g., PaiNN^29^, NequIP^25^, NewtonNet^192^, are shown to significantly increase data efficiency and predictive performance compared to models that are based on invariant convolutions. The Neural Equivariant Interatomic Potential (NequIP^25^) predicts both energy and forces utilizing E(3)-equivariant convolutions over geometric tensors. Evaluated on the MD17 data set its accuracy exceeds those of existing models while needing up to three orders of magnitude less training data. Due to its data efficiency, it was also used with coupled cluster (CCSD(T)) based training data, showing great potential for applications where a prediction accuracy beyond density functional theory (DFT) is needed. In order to further improve data efficiency, GNNFF^190^ and NewtonNet^192^ introduce more physical priors in the form of latent force vectors as well as operators containing physical information. This leads to good prediction accuracy with higher computational efficiency at only 1–10% of the training data compared to other models.

To improve generalization, hybrid models such as SpookyNet^193^ explicitly include electronic structure information such as total charge or spin state, not included in most machine-learned potentials, by applying self-attention in a transformer architecture. Empirical augmentations to include non-local and long-range contributions such as electrostatic interaction and dispersion improve transferability and at the same time enable interpretability^193^.

**GNN for large-scale MD simulations**. Many applications require models that scale to large system sizes. For example, simulations of biological systems, e.g., to study protein dynamics or drug binding mechanisms involve orders of magnitude more atoms than many other applications, while configuration changes occur on much longer timescales (10^−3^−10^3^ s) than a typical MD timestep (10^−15^ s). One way to address this enormous challenge is the development of models in a QM/MM-inspired approach, where only a small relevant subsystem, e.g., a reaction site, needs to be simulated at ab initio accuracy, while the rest of the system can be described using classical force fields.

GNNs also have the potential to support (adaptive) coarse-graining methods. They were shown to be useful in mapping atoms of a molecule into coarse-grained groups needed for large-scale simulations. The Deep Supervised Graph Partitioning Model (DSGPM)^194^ treats mapping operators as a graph segmentation problem. It predicted a coarse-grained mapping nearly indistinguishable from human annotations (see Fig. 2d). Furthermore, the machine learning framework by Wang et al.^195^, which generates a coarse-grained force field, was further improved by Husic et al.^196^ replacing manual input features with a GNN architecture making the models transferable across different molecular systems such as small proteins.

**Excited states dynamics**. GNNs were also shown as a very promising tool to tackle the challenging task of simulating excited state dynamics of complex systems^197^. Unlike ground-state dynamics, multiple potential energy surfaces as well as their crossings and couplings must be considered, leading to a higher dimensionality and complexity of the problem. Furthermore, even the generation of reliable training data using quantum mechanical calculations is challenging.

Westermayr et al. developed SchNarc^198^ for photodynamics simulations by adapting SchNet for excited states potentials, forces, and couplings, combining it with the MD framework SHARC (surface hopping including arbitrary couplings). While SchNarc is molecule specific and was applied to two compounds, CH_2_NH${}_{2}^{+}$ and CSH_2_, Axelrod et al. developed the first transferable excited state potential (see Fig. 2d)^199^. The diabatic artificial neural network (DANN) is based on PaiNN combined with a physics-informed diabatic model for photodynamic simulations for virtual screening. The resulting machine-learned potential is transferable among azobenzene derivatives and estimated to be multiple orders of magnitude faster than the underlying quantum mechanical calculation method, even considering computational effort for transfer which required additional training data.

#### Reaction prediction and retrosynthesis

While reliable property prediction and simulation methods are crucial for virtual molecular design, synthesis is often one of the main bottlenecks in the overall development process of new molecules. Progress in reaction prediction and retrosynthesis, i.e., the prediction of a reaction outcome and the design of synthetic routes for a desired product, can help to accelerate and also automate^200^ the development of new molecules. However, the two problems are still considered challenging due to the vast chemical space and currently require skills and experience from well-trained chemists. Therefore, many machine learning algorithms, e.g., seq2seq models and transformers, have been proposed for synthesis prediction and retrosynthesis, aiming at reducing manual effort. In many cases, molecules are embedded as SMILES codes, and reaction predictions as well as retrosynthesis predictions are formulated as natural language processing tasks^201–204^. Furthermore, fingerprints are widely used as structure encodings and neural networks trained on them are able to predict the most probable transformations for given molecules^205,206^. GNN based graph embeddings have recently attracted growing attention, due to the natural representation of molecular structures as graphs.

Prediction of reactivity with GNNs has been formalized into reaction center identification and graph edit tasks. Jin et al. developed a GNN-based approach to scoring each pair of connected atoms with bond change likelihood, followed by the selection of bonds with the highest score and transformation to potential products using a Weisfeiler-Lehman Difference Network (WLDN)^207,208^. The WLN architecture is also adopted by Struble and coworkers^209^ to perform multitask prediction of aromatic C-H functionalization reactions with site selectivity, while Guan et al. predict reaction outcomes with regio-selectivity through combining molecule representations learned by the WLN with on-the-fly calculated quantum mechanical descriptors^210^. In 2020, Nikitin et al. introduced a strategy to treat reaction prediction as a node classification problem, where the role of each node in the reactant graphs is predicted by a GNN with a self-attention mechanism and pseudo-global nodes^211^.

Furthermore, predicting reaction products as the result of a sequence of graph editing actions on the reactant molecules has also been investigated. One example is the work by Do et al. where graph editing actions are predicted by a reinforcement learning algorithm based on reactant and reagent molecule representations generated by GNNs^212^. In 2019, Bradshaw et al. developed a generative model that uses GNNs to predict a series of electron movements for reactions, through which the products and reaction mechanisms are predicted at the same time^213^. Apart from product prediction, GNNs are also employed to predict important reaction properties including bond dissociation energies^214,215^, transition states^216^, and activation energies^217^.

For the retrosynthesis task, recent studies can be divided into template-based and template-free approaches. The former matches the graph embedding of the product molecule to a large number of reaction templates, which determine bond changes and thus predict possible reactants, while the latter bypass the templates and directly modifies input graphs to generate synthetic precursors. Examples of template-based retrosynthesis prediction include the work by Dai et al., who predict the probability distribution of reaction templates to be fitted to the reaction outcomes by a Conditional Graph Logic Network (GLN)^218^. The reactants are generated by the GLN as the result of a probability prediction given the reaction outcome and the selected template. Another example by Ishida et al. uses GCNs to classify single retrosynthesis steps into reaction templates, where integrated gradients are applied to visualize atom contributions toward the GCN prediction^219^. More recently, Chen and coworkers proposed a framework based on MPNNs with a global attention mechanism to predict the reaction center of a target molecule, as well as the corresponding “local” reaction template based on atoms and bonds in the reaction center, which is then applied to generate reactants^220^.

For template-free retrosynthesis, Yan et al. use an edge-enhanced graph attention network to locate the reaction center in product molecules, which are transformed through bond dissociation into molecular fragments called synthons^221^. The synthon graphs are converted to SMILES and expanded to reactants by a sequence-to-sequence algorithm. At the same time, Somnath et al. proposed an approach that uses GNNs to predict a series of graph edits that transform a product into synthons that are further completed into reactants with leaving groups predicted by another GNN (see Fig. 2d)^222^. A similar strategy is adopted by Shi et al., where synthons are expanded to reactants by a variational graph translation^223^.

In 2021, Sacha et al. proposed a model that formulates both retrosynthesis and forward synthesis tasks as a sequence of graph edit actions that are predicted by an encoder-decoder structure constructed by stacking graph convolutional layers^130^.

### Crystalline and solid state systems

Compared to molecules, crystal structures and solid-state materials have some additional challenges, such as periodic boundary conditions for crystals and multiple kinds of disorder, either in form of perturbations in the crystal structure itself or in the (lack of) long-range ordering of atoms. We will present different recent applications of GNNs in solid state systems, from predicting global properties of crystal structures with and without disorder, over driving atomistic simulations, to the design of new materials aided by materials synthesis prediction, active learning, and inverse design. Table 4 gives an overview of datasets used in the following applications. We will also discuss approaches in which the trained GNN models have been analyzed to enable further insight into specific scientific questions, which is often equally important as accurate numerical predictions.

**Table 4:** Selected application-specific datasets for solid-state systems.

Dataset	Size
AFLOW^239^ - calculated properties of materials	>3,400,000
Inorganic Crystal Structure Database (ICSD)^299^ - extensive and well curated experimental database	≈210,000
Pure carbon and C-H-N-O structures at different pressures^300^	≈200,000
Materials Project^100^ - calculated properties of materials	≈145,000
Hypothetical MOF database^236^	137,953
NREL Materials Database (NRELMatDB)^301^ - computational materials database focused on renewable energy applications	≈60,000
CO and H surface binding energy dataset^302^	≈40,000
Inorganic materials synthesis recipes^303^	19,488
Perovskite structures and energies^304^	18,928
CoRE MOF database^305^ - Experimental MOF database	>14,000
bcc iron structures with energies and various kinds of defects^306^	12,193
MOF methane adsorption volume of CoRE MOFs (from GCMC)^234^	10,102
Elemental boron structures with energies^307^	5038
Computational 2D Materials Database (C2DB)^308,309^	≈4000
DDEC MOF point charges^310^	2932

#### Materials property prediction and materials design

GNNs can be used to predict a multitude of materials properties, ranging from formation energies^24,224–228^ and synthesizability prediction^229^ over band-gaps^230–232^ and other functional properties to mechanical properties^61,233^. The most straightforward application of property prediction models is the screening of large (unlabeled) crystal databases, where exhaustive screening using conventional simulation techniques (e.g., DFT) is often not feasible. Screening using GNNs only requires labels from simulation or experiment for the training set while providing fast predictions on the full database - provided the model generalizes well.

Wang et al. use a crystal GNN to predict methane adsorption volumes in metal-organic frameworks (MOFs)^234^. The pooling function leverages domain knowledge by additionally including structural properties (see Fig. 2f), e.g., the pore limiting diameter, achieving better performance than previous work^235^. They apply the model to the screening of a hypothetical MOF database by Wilmer et al.^236^ and find several high-performing candidates^234^. Gu et al. use an ensemble of attention crystal GNNs to screen alloy catalysts for CO_2_ reduction^237^. Only bulk-relaxed structures without the adsorbate (e.g., from the Materials Project (MP)^100^ database) are needed as input, removing the costly DFT relaxation from the screening process. The performance approaches that of base-line models trained on fully relaxed structures^237^.

An interesting application of GNNs was presented by Goodall et al., who use GNNs for representation learning of chemical formulas^238^. The chemical formula is represented as a dense weighted graph, in which each node corresponds to a chemical element weighted by the fraction of this element in the chemical formula. This graph representation was used to train a GNN in a supervised way to obtain a mapping from the chemical formula to an embedding. It was demonstrated that this representation has a better sample efficiency than other structure agnostic approaches.

Schmidt et al. use a crystal graph attention network for stability-screening of non-relaxed hypothetical materials, predicting the convex hull distance^24^. Only graph distances are included, making the model usable for hypothetical materials where the exact coordinates might be unknown. A vast database is used, combining MP, AFLOW^239^ and group-internal datapoints. Transfer learning then allows the screening of 15 million tetragonal perovskites of composition ABCD_2_. Dai et al. use GNNs to predict magnetostriction of polycrystalline systems (see Fig. 2g)^240^. Instead of using GNNs to model the atoms in the unit cell, individual grains and their interactions to neighboring grains are represented by nodes and edges in the GNN. This shows that GNNs can be used on different scales to predict materials properties.

Typically, screening of materials databases using ML models is only possible if training data is available which covers the target materials distribution, i.e., which adequately allows generalization to the rest of the database. Active learning offers a promising solution when the available data does not fulfill this criterion. Based on uncertainty quantification, the training dataset can then be iteratively extended to include previously uncertain data points and thereby efficiently explore the chemical space. Lu et al. use active learning to search for 2D ferromagnetic materials with a custom crystal graph multilayer descriptor in a small dataset^241^. Further applications of active learning based GNNs in materials science are promising and can be expected in the future.

#### Disordered systems and defects

Disordered materials are a wide and particularly interesting field of study, as the effects of disorder can influence or even dominate material properties. Disorder ranges from weak disorder (defects, dislocations, grain boundaries) to strong disorder (e.g., glassy systems and inhomogeneous systems such as porous materials) and includes topological/structural disorder, orientational disorder (spins, dipoles), or substitutional disorder (chemical doping, compositional disorder)^242^. Due to its inherent multi-scale nature, disorder poses severe challenges not only to materials modeling and simulation, but also to materials synthesis, characterization, and fundamental description. Graph neural networks can be extended to model various forms of disordered systems and predict their local and global properties. In contrast to quantum mechanical methods such as DFT, crystalline systems with substitutional disorder can be modeled using larger, more representative unit cells or by defining atoms/nodes with mixed occupations. Furthermore, amorphous systems can be modeled explicitly by using representative simulation boxes and aggregating over learned atom representations characteristic of the properties of the amorphous system. In the following, we will present seminal work in that direction.

One important challenge for machine learning models is how to represent substitutional disorder such as doping and fractional occupancies in the crystal graph. Chen et al. addressed this question with a MEGNet model with trainable 16-dimensional embeddings for each atom which were pre-trained on a dataset with only ordered structures^243^. The pre-trained embeddings were used to represent doped crystal sites by doing a weighted average of the elemental embeddings, weighted with (logarithmic or appropriately scaled) occupancies of the elements on the crystal sites. Additionally, it was also demonstrated how to perform multi-fidelity training with band gaps of different levels of DFT by encoding the DFT level in the global state of the MEGNet. Similarly, Wang et al. have trained a crystal GNN model on predicting the phase stability of materials (see Fig. 2f), classifying materials as metal or insulator and predicting the band gap of semiconductors^244^. They train a crystal GNN on undoped crystal structures and use this trained model to predict the properties of doped crystal structures, which they validate with DFT calculations. They find that the crystal GNN and the DFT usually predict the same trend, despite the crystal GNN not being trained on doped crystal structures^244^. Frey et al. use a crystal GNN model to predicted the properties of 2D materials with point defects (see Fig. 2f)^245^. However, the crystal GNN is only used to screen the properties of ordered structures to find promising host structures. The properties of the disordered structures were then partially calculated with DFT to train a random forest model on physics-based features which was used to screen additional structures. Another study predicted the properties of bcc iron structures with point defects^246^.

A very special application of GNNs is glassy systems. There have been works that predicted the properties of glasses^247^ and which used inverse design to find glasses with new properties^248^. Swanson et al. trained a classifier on predicting if a system is in a liquid or a glassy phase only by the positions of the atoms (see Fig. 2h)^249^. They verified that a GNN was significantly better than a CNN for this task and used self-attention to interpret the reasoning of the trained classifier. Consequently, the trained GNN was interpreted to find previously unknown relationships. Three simple and previously unknown formulas were developed, which describe the reasoning of the classifier and which could be used to differentiate the two phases. Initial work on predicting polymer properties using GNNs was based on learning representations of single monomers^250^ or of unit cells of crystalline polymers^251^.

#### Aiding simulations

Analogous to atomistic dynamics simulations of molecular systems, GNNs can also be used to simulate the dynamic behavior of crystals, i.e., to predict potential energy, forces, and partial charges, in order to drive molecular dynamics simulations. To predict forces for molecular dynamics of crystals, most approaches use conventional machine learning methods^252^, but there are first examples that use GNNs^190,253^. Raza et al. use a GNN with node-level readouts to predict partial charges of MOFs for molecular simulations of gas adsorption^254^. For each atom, a probability distribution of charge is learned and optimized globally using the maximum likelihood principle under a charge neutrality constraint. Park et al. predict forces for molecular dynamics directly using a GNN with edge-level readouts that are used to predict the magnitude of the force between atoms^190^ (see also Section ‘Molecular systems’). This avoids calculating the derivative of the potential energy and speeds up the simulation, yielding good accuracy in chemical reactions occurring at the surface of a test system of Al_2_O_3_ in contact with HF gas (see Fig. 2g). Also, good scaling accuracy transferring a model trained on a 1 × 2 × 1 supercell to a 1 × 2 × 2 supercell (Li_7_P_3_S_1_1) is achieved. This approach allows simulations of a larger scale than possible using ab initio methods with similar accuracy and might make it possible to simulate mesoscopic phenomena such as grain boundaries in the future.

#### Solid state synthesis prediction

While predicting synthesizability overall^229^ is important in the design process of new materials, predicting the products of solid state synthesis is a challenging task and interesting application area of GNNs. Malik et al. have developed a pipeline to predict the major outcome of solid-state reactions^255^. In this pipeline, GNNs were used to represent the precursors of a reaction in a set-like fashion as a dense graph while a long short-term memory layer is used to represent the series of processing actions in the reaction. Current limitations in the availability of systematic (experimental) synthesis data, including reaction conditions^11^, hinder further progress in this area. The use of electronic lab notebooks and repositories specifically designed for chemistry and materials science has a large potential to alleviate that challenge^256,257^.

#### Periodic graph generation

A major requirement for the graph-based inverse design of crystal structures is the possibility to generate new periodic graphs based on a vector representation. Recently, this problem has been addressed with a new architecture called PGD-VAE^162^, a variational autoencoder capable of generating new periodic graph structures. Another work using a VAE focuses on predicting stable crystal structures using GNNs^258^. New structures are generated in a three-step approach: First, the composition, lattice, and number of atoms of a sampled point in the latent space are decoded using a multilayer perceptron. Afterward, a random structure with these properties is assembled and a GNN trained on moving atoms to their equilibrium positions is used to generate stable structures. Despite these promising efforts, the design of solid state materials and crystal structures using GNNs is only at the beginning. Multiple challenges need to be solved to achieve wide applicability and transferability of methods to multiple classes of materials, ranging from densely packed crystals over porous materials to amorphous systems need to be solved.

## Outlook

GNNs became a very versatile and important tool very quickly. A lot has been achieved already, not only in terms of fundamental method development tailor-made for requirements of materials science and chemistry (see Section ‘Graph neural networks in materials science and chemistry’), but also in terms of applications (see Section ‘Applications’), where GNNs were successfully applied for materials simulation, screening, design, and synthesis. However, there is a wide range of open questions and challenges which need to be solved in order to leverage the full potential of GNNs in materials science and chemistry.

Despite the growing amount of research and publications on GNN model development and application, GNN models remain expert tools, i.e., they are comparably hard to implement, adapt, transfer, train, and apply to given datasets and applications. Libraries such as PyTorch Geometric^259^, DGL^260^ or the Keras based KGCNN^261^ implement a selection of state-of-the-art GNN layers and models. However, the use of such libraries in many cases requires expert knowledge which goes beyond the knowledge needed for the successful application of more established machine learning models. One of the reasons for this is certainly related to the fast development of new GNN variants which are partially hard to compare. The widespread use and further development of common benchmarks (e.g., the QM9 dataset) as well as open communication of models and training workflows, including open-source code, inspired by common practice in the machine learning community, are essential for a reliable quantitative comparison of future developments. However, non-optimal hyperparameters and the (prohibitively) high computational cost of hyperparameter optimization is an open challenge. A further challenge hindering the transfer of state-of-the-art models to applications is the discrepancy in the data distribution between widely used benchmark datasets (e.g., QM9) and actually relevant datasets, which typically contain larger molecules (i.e., larger graphs), which are more diverse, e.g., in terms of the chemical elements used and in the size variance, and which sample the chemical or materials space less densely then QM9^262^.

Generally, there is more research and model development activity for molecules and chemistry, compared to materials science. As a consequence, the transfer of existing GNNs to new application areas (e.g., porous or amorphous materials) might be challenging and requires further development of GNN models. To some extent, this can be attributed to a lack of generic benchmark datasets for crystalline materials and, even more importantly, (partially) disordered structures and amorphous materials. OC20 is one of the few examples of datasets covering both materials science and chemistry^102^. Nonetheless, there are many promising application areas of GNNs for solid-state materials. In contrast to quantum mechanical simulation methods such as DFT, GNNs are particularly suited for representing disordered systems and predicting their properties, e.g., systems with compositional disorder such as high-entropy alloys. However, there is a lack of large datasets of disordered systems, particularly labeled datasets with consistent measured or simulated properties. Overall, screening of (hypothetical) materials spaces with predictive ML models promises the rapid discovery of new materials with optimized target properties, especially when combined with transfer and active learning to improve data efficiency and reduce computational cost.

The first steps towards GNN-based generative models exist, but there are many open challenges of reliability and transferability. Despite the high potential of GNN-based algorithms for the inverse design of new molecules and materials, convincing success stories of ML-designed materials are rare. To make generative models more application-relevant, new methods are required that e.g., allow to include constraints in the design process, in the simplest case symmetries of generated molecules and materials, or in more complex scenarios additional (empirical or analytical) objectives such as synthesizability. A large step in that direction is new representations, not only for organic molecules but also for (3D) materials^263–265^.

Finally, more research on the explainability and interpretability of GNNs and machine learning in general will help to better understand underlying correlations and eventually causal relations in large and complex datasets, eventually contributing to scientific understanding and progress^266–268^.
